# Comparison of four channelled videolaryngoscopes to Macintosh laryngoscope for simulated intubation of critically ill patients: the randomized MACMAN2 trial

**DOI:** 10.1186/s13613-021-00916-3

**Published:** 2021-08-16

**Authors:** Paul Decamps, Nicolas Grillot, Aurelie Le Thuaut, Noelle Brule, Corinne Lejus-Bourdeau, Jean Reignier, Jean-Baptiste Lascarrou

**Affiliations:** 1 Médecine Intensive Réanimation, Centre Hospitalier Universitaire de Nantes, Nantes, France; 2 Service d’Anesthésie Réanimation Chirurgicale, Université de Nantes, CHU Nantes, Pôle Anesthésie-Réanimation, Hôtel Dieu, 44093 Nantes, France; 3 Laboratoire Expérimental de Simulation de Médecine Intensive de L’Université (LE SiMU) de Nantes, 9 rue Bias, 44001 Nantes, France; 4 Plateforme de Méthodologie Et Biostatistique, Direction de La Recherche de L’Innovation, Centre Hospitalier Universitaire de Nantes, Nantes, France; 5 Médecine Intensive Réanimation, Centre Hospitalier Universitaire de Nantes, Nantes, France

**Keywords:** Intensive care, Macintosh laryngoscope, Videolaryngoscope, Endotracheal intubation, High-fidelity simulation

## Abstract

**Background:**

Videolaryngoscopes with an operating channel may improve the intubation success rate in critically ill patients. We aimed to compare four channelled videolaryngoscopes to the Macintosh laryngoscope used for intubation of a high-fidelity simulation mannikin, in a scenario that simulated critical illness due to acute respiratory failure.

**Results:**

Of the 79 residents who participated, 54 were considered inexperienced with orotracheal intubation. Each participant used all five devices in random order. The first-pass success rate was 97.5% [95% CI 91.1–99.7] for Airtraq™, KingVision™, and Pentax AWS200™, 92.4% [95% CI 84.2–97.2] for VividTrac VT-A100™, and 70.9% [95% CI 59.6–80.6] for direct Macintosh laryngoscopy. The first-pass success rate was significantly lower with direct Macintosh laryngoscopy than with the videolaryngoscopes (*p * <  0.0001 for Airtraq™, KingVision™, Pentax AWS200™, and VividTrac VT-A100™).

**Conclusion:**

The Airtraq™, KingVision™, and Pentax AWS200™ channelled videolaryngoscopes produced high first-pass success rates with a lower boundary of the 95% CI above 90%. A multicentre, randomised controlled clinical study comparing channelled videolaryngoscopy to direct laryngoscopy should include one of these three videolaryngoscopes.

**Supplementary Information:**

The online version contains supplementary material available at 10.1186/s13613-021-00916-3.

## Introduction

Endotracheal intubation (ETI) in critically ill patients is a risky procedure associated with serious complications in up to 25–40% of cases [1–3] and with cardiac arrest in 1.6–2.7% of cases [4]. The main determinant of complications is failure of the first ETI attempt. The number of ETI attempts, i.e., of laryngoscopies, correlates with the risk of serious complications [5–7]. Difficult ETI defined as two failed orotracheal intubations under laryngoscopy guidance is far more common in the intensive care unit (ICU) than in the operating room, with reported frequencies ranging from 10 to 20% [8–11].

Videolaryngoscopes were developed to facilitate orotracheal intubation. These devices allow complete indirect vision of the glottis even when the oro-pharyngo-laryngeal axis is misaligned. Although their role in the intensive care unit (ICU) remains unclear [12], they are now available in about 75% of ICUs [13, 14]. However, their use as the first-line ETI tool ranges from over 90% of cases in the US [15] to less than 5% of cases in France [13]. This variability is related to the absence of convincing evidence that videolaryngoscopy improves first-attempt success rates in the ICU: several studies compared first-line videolaryngoscopy to first-line direct laryngoscopy using a Macintosh blade [10, 16–19], but meta-analyses of their findings were inconclusive [20–22]. In the MACMAN1 randomised controlled trial of videolaryngoscopy vs. Macintosh direct laryngoscopy [10], the main cause of ETI failure in the videolaryngoscopy group was glottis catheterisation failure (70.7%), which occurred despite clear and complete visualisation of the glottis. Videolaryngoscopes that have an operating channel to guide the tube into the trachea, of which several models are available, should reduce the risk of catheterisation failure. However, few studies have compared the various models, and most of them were done in patients undergoing elective surgery or using low-fidelity dummies. The Pentax AWS200™ and KingVision™ proved more efficient than the standard videolaryngoscope during ETI for cardiac arrest [23, 24], whereas another study found no significant differences [25]. The A.P. Advance™ Difficult airway blade was inferior to direct laryngoscopy and to other videolaryngoscopes in a study of elective-surgery patients equipped with a collar to simulate a difficult airway [26]. This variability in results is probably ascribable to differences in device design.

The objective of this randomised controlled trial (MACMAN 2 trial) was to evaluate four channelled videolaryngoscopes (KingVision™, Airtraq™, VividTrac VTA-100™, and Pentax AWS200™) in a realistic ICU scenario of orotracheal intubation on a high-fidelity mannikin. Our main hypothesis was that each channelled videolaryngoscope had a lower 95% confidence interval boundary greater than 90%.

## Methods

### Design

The MACMAN 2 trial used a randomised, open-label, single-centre design. Orotracheal intubation was performed on a high-fidelity simulator. The study was registered on As predicted before inclusion of the first participant (#15045).

### Participants

Recruitment was carried out among anaesthesia and intensive-care residents in their first and second years, emergency-medicine residents in their first to third years, and medical specialty residents who had completed at least one ICU rotation. The participants were contacted by e-mail via existing mailing lists for the different specialties.

The participants were categorised as experienced or inexperienced based on whether they had performed 50 or more successful ETIs using direct laryngoscopy. Before the study, all participants attended a theoretical class that included an 8-min video explaining the study objective and describing the four videolaryngoscopes and their modalities of use according to the manufacturers’ recommendations. The video also showed ETI using a laryngoscope equipped with a Macintosh blade.

### Intubations on the high-fidelity simulator

The orotracheal protocol has been described elsewhere [10, 27]. It consisted of a realistic scenario of ETI in an ICU patient with acute respiratory failure. The study participants performed ETIs on a high-fidelity simulator (SimMan3G™, Laerdal Medical, Stavanger, Norway). One of the investigators (i.e., an intensivist with extensive ETI experience) was also present and played the role of the on-duty supervising senior intensivist. ETI was made more difficult by inflating the tongue to reduce the size of the pharynx, thereby impairing visualisation of the glottis.

Pre-oxygenation was ensured by a self-refilling balloon with a unidirectional valve (bag-valve-mask, BVM) connected to wall oxygen and fed by a minimum of 15 L/min for a minimum of 3 min [28, 29]. Fictitious anaesthetic induction was then performed by combining a hypnotic agent (etomidate) and a neuromuscular blocking agent (succinylcholine), in accordance with international [30] and French [31] guidelines.

**Fig. 1:**
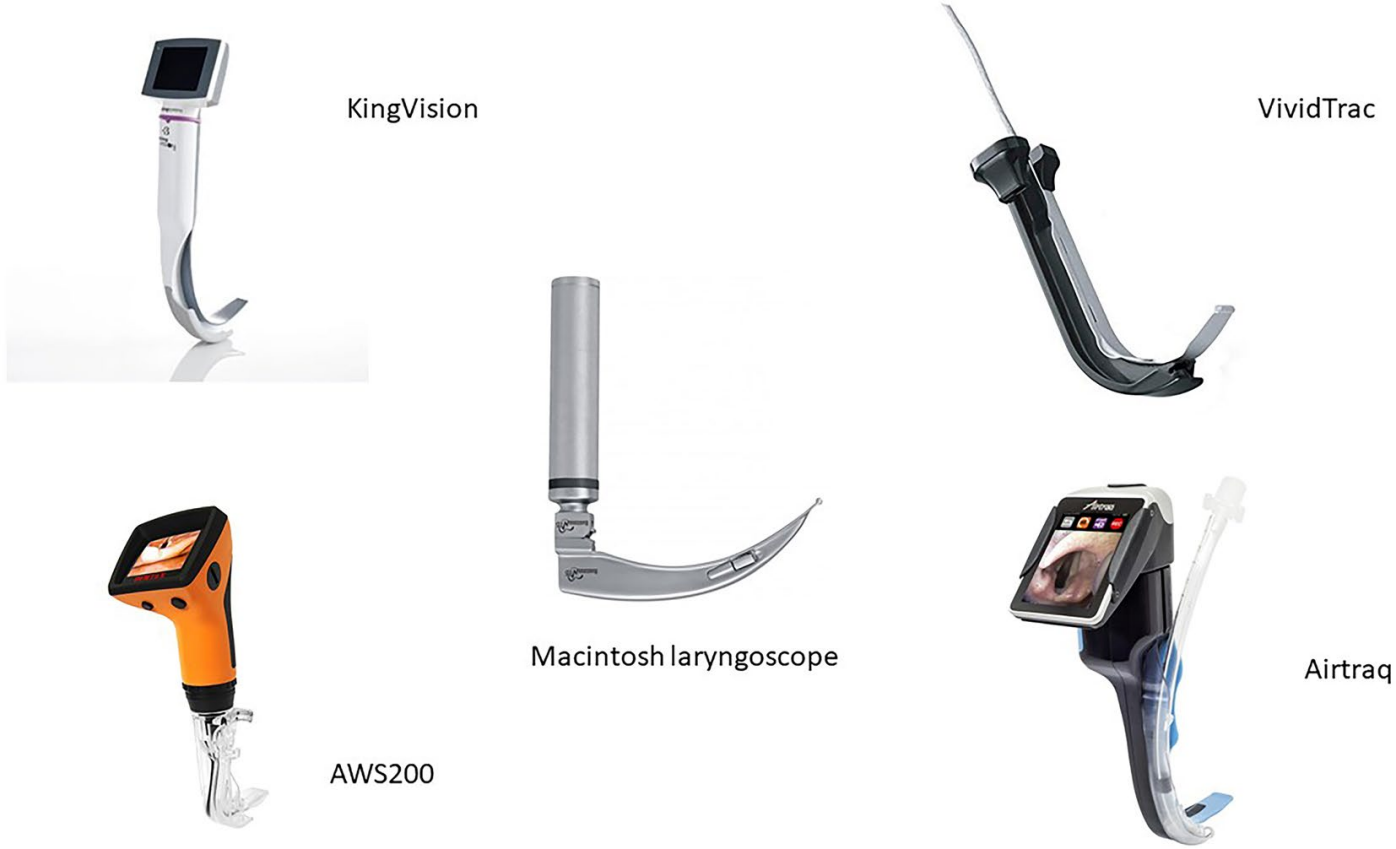
Videolaryngoscopes used in the MACMAN2 trial

Laryngoscopy was performed using the randomly assigned device (Fig. 1). The five devices were KingVision™ (AMBU, Bordeaux, France), AWS200™ (PENTAX, Argenteuil, France), Airtraq™ (VYGON, Ecouen, France), VividTrac VT-A100™ (VIVID, Palo Alto, CA), and a Macintosh size 3 blade for direct laryngoscopy. A size 8 PORTEX™ tube (Smiths Medical France, Rungis, France) was introduced. If intubation proved difficult, the participant could ask for a bougie, without this being considered an intubation failure. The tube balloon was inflated then manually ventilated using a BVM. The intra-tracheal position of the tube was established based on the capnography curve over more than three breathing cycles.

If the first ETI attempt failed, i.e., if the videolaryngoscope or Macintosh blade was removed from the mouth of the mannikin without the tube being in the trachea, the investigator encouraged the participant to try again. The participant could choose to request a backwards, upwards, and rightwards pressure (BURP) manoeuvre or to use a bougie for this second attempt. BVM insufflation was not permitted before or after the first laryngoscopy even if desaturation occurred, because the mannikin was not able to detect successful intubation after BVM insufflation.

Laryngoscopy duration was defined as the time from the beginning of mannikin fasciculations to confirmation of the intratracheal position of the tube based on the first inflection of the expired CO_2_ curve. The desaturation model used was constructed from clinical monitoring data collected during the MACMAN study [10].

The participant then repeated this procedure using the other four devices in the order assigned at random. The simulation scenario was re-started at the beginning of the use of each device.

At the end of the session the participant was asked to rate the ease of use of each device.

### Randomisation

Before each session for each participant, the order in which the five devices were used was drawn at random by the investigator by interrogating a dedicated website (https://nantes-lrsy.hugo-online.fr/EnnovClinical/). The randomisation sequence was built by a statistician who was not otherwise involved in the trial (ALT). Blinding was not feasible.

### Outcomes

The primary outcome was the first-pass success rate with each device [32]. ETI failure was defined as oesophageal intubation or removal of the videolaryngoscope or Macintosh blade from the mouth of the mannikin.

The secondary outcomes were time to successful intubation, the Cormack–Lehane and POGO glottis visibility scores, the proportion of attempts resulting in oesophageal intubation, the proportion of attempts resulting in selective bronchial intubation, the median lowest SpO_2_ value computed from the desaturation model, and ease of device use rated by the participants on a scale from 0 (not at all easy) to 10 (very easy).

### Ethics

The study protocol was approved by the Ethics Committee of the Société de Réanimation de Langue Française (CE SRLF 17–11). Each participant was informed at the beginning of each session about the objectives of the trial, the recording of the simulation sessions, and her or his right to access and refuse the use of the data collected for the trial.

### Statistics

Based on a previous study, we defined a 90% first-attempt success rate as the lowest clinically acceptable rate for a device designed for the management of difficult airways [26]. We, therefore, defined the target first-attempt success rate as 95% for the channelled videolaryngoscopes, with a lower boundary of the 95% confidence interval (95% CI) of 90% or more. With the alpha risk set at 5%, to obtain 80% power we needed 73 intubations using each device. To account for possible missing data, we decided to include 80 intubations per device.

The statistical analysis was performed using SAS^®^ software (SAS Institute, Cary, NC) with the intention-to-treat approach (each intubation was analysed in the group assigned at random). The participants were described by the number and percentage of each modality for qualitative variables and by the mean  ±  SD, range, and quartiles for quantitative variables.

To assess the primary outcome, we chose the Chi-square test to compare the five groups.

The secondary outcomes were evaluated using mixed generalised linear or logistic regression models depending on the variable type.

*P* values were considered significant if  <  0.05 for the primary outcome. For the secondary outcomes, Bonferroni’s correction was applied to the comparisons of the four videolaryngoscopes to the Macintosh laryngoscope, and significant *p* values were  <  0.006.

## Results

Between September 2017 and March 2020, we included 79 residents (Additional file 1: Figure S1), including 40 (51%) in intensive care or anaesthesiology (Table 1). Of the 79 residents, 75 (96%) had already performed at least one ETI on a patient by direct laryngoscopy with a Macintosh blade (75 out of 79; 96%) and 64 (81%) had already used a videolaryngoscope on a patient. The criterion for being an experienced intubator was met by 25 (32%) participants.

**Table 1:** Participant characteristics

Characteristics	Description (*n * = 79)
Area of training, *n* (%)	
Emergency medicine	24 (30%)
Anesthesiology and intensive care	40 (51%)
Other	15 (19%)
Number of semesters of residency, median [IQR]	3 [1; 5]
Prior experience with direct laryngoscopy on a manikin	
0	4 (5%)
1–4	24 (31%)
5–9	31 (40%)
10–19	18 (23%)
≥ 20	1 (1%)
Prior experience with direct laryngoscopy on patients	
Qualified as non-expert	
0	4 (5%)
1–4	12 (15%)
5–9	6 (8%)
10–19	16 (20%)
20–49	16 (20%)
Qualified as Expert	
≥ 50	25 (32%)
Prior experience with videolaryngoscopy on a mannikin	
None	20 (25%)
≥ 1	59 (75%)
Prior experience with videolaryngoscopy on patients	
None	33 (42%)
≥ 1	46 (58%)
Prior experience with videolaryngoscopy without a guiding channel on patients	
0	40 (51%)
1–4	19 (24%)
5–9	10 (13%)
10–19	8 (10.1%)
≥ 20	2 (2.5%)
Prior experience with videolaryngoscopy with a guiding channel on patients	
0	52 (66%)
1–4	21 (26.6%)
5–9	3 (3.8%)
10–19	3 (3.8%)
≥ 20	0 (0%)

### Primary outcome

The first-attempt success rate was 97.5% (95% CI 91.1–99.7) for Airtraq, KingVision, and Pentax; 92.4% [95% CI 84.2–97.2] for VividTrac; and 70.9% [95% CI 59.6–80.6] for direct laryngoscopy. The lower boundary of the 95% CI was greater than 90% for three devices, the exceptions being VividTrac and direct laryngoscopy.

### Secondary outcomes

#### First-pass success

**Fig. 2:**
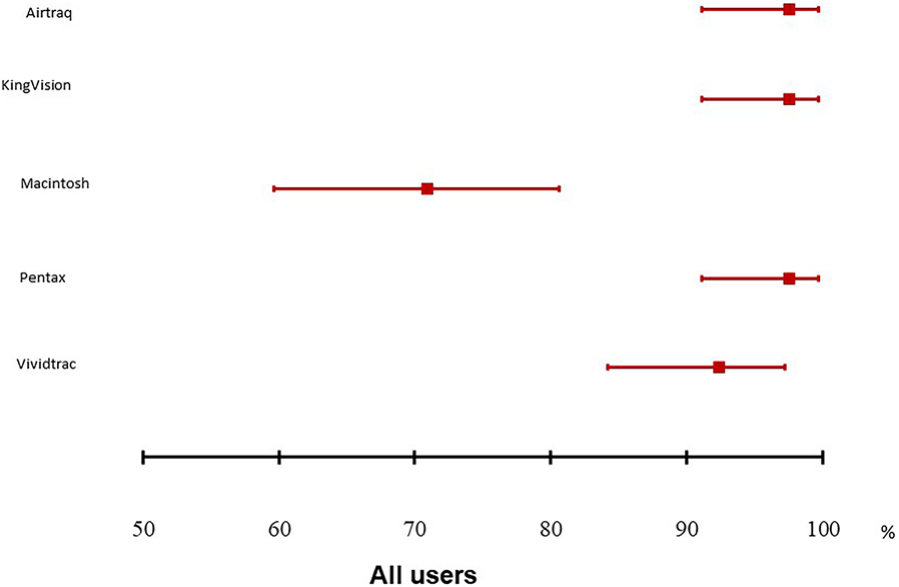
Forest plot of the first-pass success rate with each videolaryngoscope included in the MACMAN2 trial

There were no significant differences between the first-attempt success rate with VividTrac and with each of the other three videolaryngoscopes (92.4 vs. 97.5%; *p * =  0.18, *p * =  0.20, and *p*  =  0.38, respectively; Fig. 2). The first-attempt success rate for direct laryngoscopy was significantly lower compared to those for the Airtraq, KingVision, and Pentax AWS200 (70.9 vs. 97.5%; *p * <  0.0001; Fig. 2) and to that for the VividTrac (70.9 vs. 92.4%; *p * <  0.0001). Experienced and inexperienced intubators obtained similar results (Additional file 2: Figure S2; Additional file 3: Figure S3).

#### Intubation time

The laryngoscopy time was longer with direct laryngoscopy than with the Airtraq, KingVision, and Pentax AWS200 (92.7 s vs. 67.7 s, 66.4 s, and 62.8 s, respectively; *p * <  0.001, *p * <  0.001, and *p * <  0.001, respectively). It was also longer with VividTrac than with Pentax AWS200™ (82.9 vs. 62.8 s; *p * =  0.0028). There was no difference in laryngoscopy time between direct laryngoscopy and VividTrac (92.7 vs. 82.9 s; *p * =  0.12).

#### Glottic visualisation

Glottic visualisation, as assessed by the POGO score, was poorer with direct laryngoscopy than with the four videolaryngoscopes (57.1 vs. 85.5, 91.0, 89.7 and 79.3 for Airtraq, KingVision, Pentax AWS200, and VividTrac, respectively; *p * <  0.001 for all four comparisons). The results were similar when the experience of the participants was taken into account (Table 3). Glottic visualisation was poorer with VividTrac compared to KingVision and Pentax AWS200 (79.3 vs. 91.0 and 89.7, respectively; *p * <  0.0001 and *p * =  0.0003, respectively).

#### Ease of use

Ease of use of the device as rated by the participants was lower with the Macintosh blade than with Airtraq™, KingVision™, and Pentax AWS200™ (6.35 vs. 7.09, 7.41, and 7.93, respectively; *p * <  0.0001 for all three comparisons, Table 2). There was no difference between the Macintosh blade and VividTrac (6.35 vs. 6.20; *p * =  0.66). Again, the results were similar when the experience of the participants was taken into account (Table 3).

**Table 2:** Comparison of the five devices

	Macintosh *n* = 79	AirTraq *n* = 79	KingVision *n* = 79	Pentax AWS200 *n* = 79	VividTrac *n* = 79	*p* value
First-pass success	56 (70.8%)	77 (97.5%)	77 (97.5%)	77 (97.5%)	73 (92.4%)	< 0.001
Number of attempts before success						
1	56 (70.8%)	77 (97.5%)	77 (97.5%)	77 (97.5%)	73 (92.4%)	< 0.001
2	11 (14.0%)	0 (0%)	2 (2.5%)	2 (2.5%)	1 (1.3%)	
3	11 (14.0%)	1 (1.3%)	0 (0%)	0 (0%)	4 (6.3%)	
4	1 (1.2%)	0 (0%)	0 (0%)	0 (0%)	0 (0%)	
Time to intubation, s, mean ± SD	92.7 ± 59.8	67.7 ± 30.7	66.4 ± 24.1	62.8 ± 33.9	82.9 ± 42.8	< 0.001
Lowest SpO_2_, median [IQR]	86 [81–89]	88 [87–90]	88 [86–89]	89 [87–90]	86 [85–89]	^$^
Bougie use	32 (40.5%)	0 (0%)	0 (0%)	0 (0%)	0 (0%)	< 0.001
BURP manoeuvre	18 (22.8%)	0 (0%)	0 (0%)	0 (0%)	1 (1.3%)	*
Percentage glottic opening, mean ± SD	57.1 ± 25.9%	85.5 ± 15.2%	91.0 ± 13.3%	89.7 ± 13.5%	79.3 ± 20.9%	< 0.001
Cormack–Lehane grade						
1	23 (30%)	69 (90%)	72 (95%)	71 (91%)	62 (80%)	< 0.001
2	39 (51%)	8 (10%)	4 (5%)	7 (9%)	15 (19%)	
3	13 (16%)	0 (0%)	0 (0%)	0 (0%)	0 (0%)	
4	2 (3%)	0 (0%)	0 (0%)	0 (0%)	1 (1%)	
Ease of device use^a^	6.35 ± 2.20	7.09 ± 1.59	7.41 ± 1.59	7.93 ± 1.47	6.20 ± 2.05	< 0.001
Oesophageal intubation	10 (12.66%)	1 (1.27%)	1 (1.28%)	0 (0%)	0 (0%)	*
Selective intubation	6 (7.59%)	2 (2.53%)	3 (3.8%)	5 (6.3%)	5 (6.3%)	*
Blade placed under the epiglottis	8 (10%)	56 (71%)	29 (37%)	79 (100%)	64 (81%)	< 0.001

The data are the mean  ±  SD or number (%)

*BURP* backwards, upwards, and rightwards pressure applied to the larynx

^a^Rated by the participants on a scale from 0 to 10

^*^The number of events was too small to allow a meaningful analysis

^$^Not compared as SpO_2_ correlates with intubation time in a mannikin study

**Table 3:** Comparison of the five devices used by participants who were inexperienced (*n*  =  54) vs. experienced (*n*  =  25) with orotracheal intubation in critical care

Groups	Macintosh *n* = 79	AirTraq *n* = 79	KingVision *n* = 79	Pentax AWS200 *n* = 79	VividTrac *n* = 79	*p* value
First-attempt success						
Experienced	18 (72.0)	25 (100.0)	25 (100.0)	24 (96.0)	23 (92.0)	< 0.001
Inexperienced	38 (70.0)	52 (96.3)	52 (96.3)	53 (98.2)	50 (92.6)	< 0.001
Time to intubation (s)						
Experienced	86.73 (57.3)	55.41 (16.4)	56.55 (25.5)	50.44 (20.7)	78.16 (45.8)	< 0.001
Inexperienced	95.46 (61.3)	73.56 (34.2)	70.97 (22.3)	68.52 (37.4)	85.14 (41.5)	0.002
Lowest SpO_2_ (%)						
Experienced	85.33 (5.21)	88.09 (1.78)	88.13 (2.87)	88.74 (2.14)	87.13 (4.36)	0.01
Inexperienced	84.02 (7.39)	86.88 (3.74)	87.12 (2.14)	87.48 (3.75)	85.70 (3.75)	< 0.001
Percentage of glottic opening						
Experienced	56.88 (29.4)	88.96 (13.0)	90.42 (13.9)	88.54 (13.6)	76.04 (19.6)	< 0.001
Inexperienced	57.25 (24.4)	83.98 (15.9)	91.30 (13.2)	90.19 (13.6)	80.74 (21.4)	< 0.001
Ease of device use						
Experienced	6.92 (1.93)	7.29 (1.73)	7.83 (1.63)	8.00 (1.79)	5.67 (1.74)	< 0.001
Inexperienced	6.08 (2.29)	7.00 (1.53)	7.21 (1.55)	7.90 (1.32)	6.44 (2.15)	< 0.001

The data are the mean  ±  SD or number (%)

^a^Rated by the participants on a scale from 0 to 10

## Discussion

In this study, three of the four videolaryngoscopes, KingVision™, Pentax AWS200™, and Airtraq™, met our predefined target of a lower 95% CI boundary of 90% or more for the proportion of successful first-pass attempts. With all four videolaryngoscopes, the first-pass success rate was significantly higher than with direct laryngoscopy using a Macintosh blade.

A comparative mannikin study found that videolaryngoscopy with a Macintosh-like blade performed better than did channelled videolaryngoscopy for the normal airway, whereas the opposite was true for difficult airways [32]. In a study that used a mannikin with a normal airway, direct laryngoscopy was fastest and also provided a higher first-pass success rate than did the Airtraq™ in the hands of non-experts [33]. These differences with our results are due to the lower proportion of first-pass success with direct laryngoscopy intubation in our trial compared to previous studies: first-pass success rates ranged from 79 to 100% with direct laryngoscopy, even in the hands of non-experts, [32–35] compared to 71% in our study. The first-pass success rates for the videolaryngoscopes, in contrast, were comparable, at about 83–100% [32–34]. We suggest several hypotheses to explain these differences. First, intubation was made more difficult in our study by inflating the tongue to reduce the size of the pharynx and to impair glottic visualisation. We made this choice, because the proportion of successful first-pass intubations with videolaryngoscopy performed by inexperienced operators on ICU patients was 67.7% in the MACMAN1 trial [10]. Two of the above-mentioned studies on mannikins used only a normal airway [33, 35]. The mean POGO score by direct laryngoscopy ranged from 77 to 80 in the two studies reporting this variable [32, 34], both of which simulated difficult airways, compared with 57 in our study. Second, the use of a stylet or bougie to guide the tube was not allowed in our study for the first attempt, despite the simulated difficult airway. Using a stylet or bougie is recommended for the first attempt when the intubation is predicted to be difficult and secondarily if the first attempt fails [31]. For direct laryngoscopy intubation, a recent study found that a bougie was superior over a short stylet [15] and another study that a stylet was superior over no stylet [37]. If the first attempt failed, the participant could request a bougie in the present study, as happened for 40.5% of the direct laryngoscopy intubations. Finally, the previous studies did not recreate a scenario that replicated intubation of a critically ill patient in the ICU. We were able to use data from the MACMAN1 trial to replicate the conditions of intubation in critical care, which are associated with an increased frequency of complications and a decreased first-pass success rate.

Few studies have compared different types of channelled videolaryngoscope. Outside the setting of critical care, a study of 720 patients undergoing elective surgery used a cervical collar to increase intubation difficulty. All intubations were performed by senior anaesthesiologists. Three channelled videolaryngoscopes were used: Airtraq and KingVision were associated with first-pass success rates of 85 and 87%, respectively, compared to only 37% with A.P. Advance™ [26]. We found lower first-pass success rates with VividTrac compared to the other three videolaryngoscopes, although the difference was not statistically significant. In another study, the intubation time was longer with VividTrac than with KingVision [36]. In a comparison of KingVision, Airtraq, and VividTrac that used an airway trainer with normal and difficult airway options and included medical students with no intubation experience, the only significant difference was a higher first-pass success rate with VividTrac than with KingVision for the difficult airway (100 vs. 92%, respectively; *p * <  0.05) [34]. In contrast, in our study, VividTrac was the only channelled videolaryngoscope for which the lower boundary of the 95% CI for the first-pass success rate was below 90%. Furthermore, the duration of laryngoscopy was significantly longer and glottic visualisation poorer compared to the other three videolaryngoscopes. The participants also gave the VividTrac lower ease-of-use ratings compared to the other devices.

In our study, the first-pass success rate was significantly higher with the videolaryngoscopes than with direct laryngoscopy for both experienced and inexperienced intubators. The differences between the videolaryngoscopes and direct laryngoscopy in terms of laryngoscopy time, glottic visualisation, and ease of use were also similar in the two participant groups. This finding is somewhat surprising, since a higher level of experience with intubation has been shown to correlate with a higher first-pass success rate when performing emergency ETI in critically ill patients [37, 38]. Furthermore, in a metaanalysis focussing on ETI outside the operating room, first-pass intubation was more common with a videolaryngoscope than with direct laryngoscopy among the novices (81.4 vs. 71.5%; odds ratio, 1.95; 95% CI, 1.45–2.64; *p * <  0.001) but not among the experienced intubators [20]. However, novices were variably defined in the different studies included in this work. The shorter learning curve for videolaryngoscopy than for direct laryngoscopy may explain that novices do better with the former [39]. Thus, having performed only 15 or more videolaryngoscopies was independently associated with first-pass success in critically ill patients [39]. We used 50 intubations as the cutoff to differentiate inexperienced from experienced intubators based on a meta-analysis showing that, above this level of experience, the success rate of one or two attempts was greater than 90% [38]. Most of our participants had limited experience with intubation, and the residents most likely to acquire such experience, i.e., those in emergency medicine and intensive care, were only in their first or second year of residency. All participants would have met the definition of non-experts used in the MACMAN trial (5 years in ICUs or 2 years in anaesthesia and 1 year in ICUs) [38]. We selected these participants, because novice intubators perform the first intubation attempt in more than 80% of patients in ICUs in France [10].

Our trial has several limitations. First, extrapolation from results obtained using a mannikin to those in patients requires circumspection. Airway anatomy is among the major determinants of intubation difficulty and, despite recent advances, mannikins do not accurately replicate the human airway anatomy. For instance, by computed tomography, the upper airway size of the SimMan 3G^®^ was larger than that of patients [40]. Soft tissue elasticity is an important feature during direct laryngoscopy and was not accurately replicated by our mannikin. Other details were not present in the simulation, such as fogging or secretions that may limit visibility during intubation. However, it is ethically preferable to perform this type of study on mannikins [41], thus protecting patients from potential complications related to videolaryngoscopy use by novices. High-fidelity simulation also makes it possible to create a difficult airway, which is not extraordinarily frequent in clinical practice, and ensures that the study environment remains unchanged for all participants and all devices[42, 43]. Second, oxygenation in the event of desaturation was not technically feasible with our setup. This probably overestimated the first-pass success rate as, in the event of a long intubation time, the participant did not stop the attempt to re-ventilate the patient. There was also no possibility of changing the intubation device for another possibly more effective one. This point no doubt increased the mean intubation time. However, the mean intubation time, which was the laryngoscopy time increased by 45 s to allow for induction, was lower than that found in the MACMAN 1 trial [10]. Last, we defined experts as having performed more than 50 direct laryngoscopies. This level of experience in direct laryngoscopy may not translate to a similar level with videolaryngoscopy. In an observational ICU study reported in 2020, experts were defined as having performed at least 15 videolaryngoscopy ETIs [41].

## Conclusion

Among the four videolaryngoscopes tested, three had a lower boundary of the 95% CI for the first-pass success rate higher than 90%: Airtraq™, KingVision™, and Pentax AWS200™. These three devices were superior over direct laryngoscopy in our model of a patient in critical condition due to acute respiratory failure. Further randomised controlled trials are required to better define the role for videolaryngoscopes and associated tools for the intubation of critically ill patients. Including the three videolaryngoscopes found efficient in the present study, and pooling them due to their similar performance, may facilitate such trials.

## Supplementary Information

**Additional file 1: Figure S1.** Consort flow diagram template.

**Additional file 2: Figure S2.** Inexperienced users.

**Additional file 3: Figure S3.** Experienced users.

## Data Availability

The study data will be made available upon reasonable request to the corresponding author.
