# Supplementary material for: Comparison of four channelled videolaryngoscopes to Macintosh laryngoscope for simulated intubation of critically ill patients: the randomized MACMAN2 trial
Source: Ann Intensive Care. 2021 Aug 16;11:126. doi: 10.1186/s13613-021-00916-3 (PMC8368860; doi:10.1186/s13613-021-00916-3)
Supplement: Supplementary file 1 — Additional file 1: Figure S1. Consort flow diagram template. [file 13613_2021_916_MOESM1_ESM.doc]

Assessed for eligibility

(n = 79)

**Enrollment**

Excluded (n = 0)

Randomised (n = 79)

#

**Allocation**

**Follow-up**

**Analysis**

Allocated to intervention

(n = 79)

Received allocated intervention (n = 79)

Did not receive allocated intervention (n = 0)

Lost to follow-up

(n = 0)

Discontinued intervention (n = 0)

Analysed (n = 79)

Excluded from analysis

(n = 0)
