# Supplementary figures and images for: Comparison of four channelled videolaryngoscopes to Macintosh laryngoscope for simulated intubation of critically ill patients: the randomized MACMAN2 trial
Source: Ann Intensive Care. 2021 Aug 16;11:126. doi: 10.1186/s13613-021-00916-3 (PMC8368860; doi:10.1186/s13613-021-00916-3)

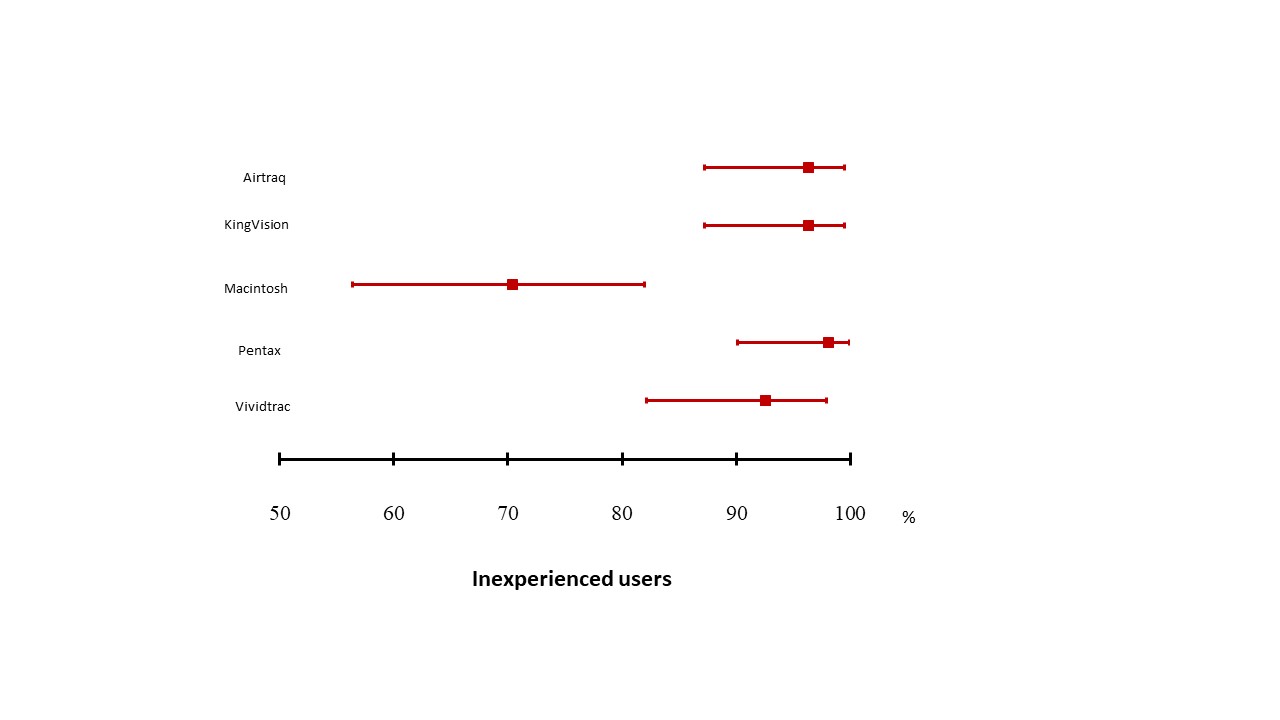

Supplement: Supplementary file 2 — Additional file 2: Figure S2. Inexperienced users. [file 13613_2021_916_MOESM2_ESM.jpg]

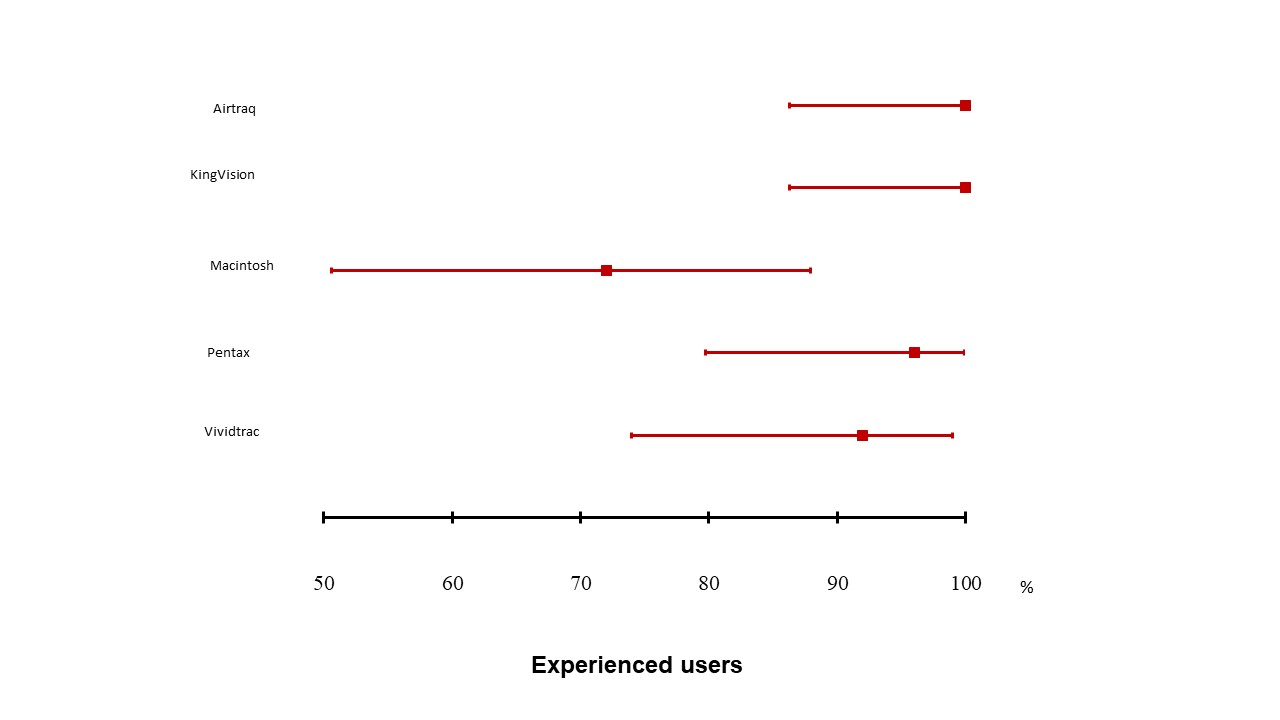

Supplement: Supplementary file 3 — Additional file 3: Figure S3. Experienced users. [file 13613_2021_916_MOESM3_ESM.jpg]
